# Generation of hydroxyl radicals by Fe-polyphenol-activated CaO_2_ as a potential treatment for soil-borne diseases

**DOI:** 10.1038/s41598-018-28078-6

**Published:** 2018-06-27

**Authors:** Cláudio Kendi Morikawa

**Affiliations:** National Agriculture and Food Research Organization, Division of Vegetable Pest Management and Functional Analysis, Institute of Vegetable and Floriculture Science, 514–2392, Ano, Kusawa 360, Mie Tsu, Japan

## Abstract

An Fe-polyphenol catalyst was recently developed using anhydrous iron (III) chloride and coffee grounds as raw materials. The present study aims to test the application of this Fe-polyphenol catalyst with two hydrogen peroxide (H_2_O_2_) sources in soil as a new method for controlling the soil-borne disease caused by *Ralstonia solanacearum* and to test the hypothesis that hydroxyl radicals are involved in the catalytic process. Tomato cv. Momotaro was used as the test species. The results showed that powdered CaO_2_ (16% W/W) is a more effective H_2_O_2_ source for controlling bacterial wilt disease than liquid H_2_O_2_ (35% W/W) when applied with an Fe-polyphenol catalyst. An electron paramagnetic resonance spin trapping method using a 5,5-dimethyl-1-pyrroline-N-oxide (DMPO) assay and Fe-caffeic acid and Fe-chlorogenic acid complexes as models showed that these organometallic complexes react with the H_2_O_2_ released by CaO_2_, producing hydroxyl radicals in a manner that is consistent with the proposed catalytic process. The application of Fe-polyphenol with powdered CaO_2_ to soil could be a new environmentally friendly method for controlling soil-borne diseases.

## Introduction

*Ralstonia solanacearum* is one of the top ten most scientifically and economically important bacterial species related to plant diseases^1^. This disease causes bacterial wilt in papayas (*Carica papaya*), potatoes (*Solanum tuberosum*), tomatoes (*Lycopersicum esculentum*), eggplant (*Solanum melongena*), bananas (*Musa* spp) and groundnuts (*Arachis hypogaea*)^2^ and causes serious economic losses worldwide^3^.

Bacterial wilt in vegetable crops induced by *Ralstonia solanacearum* is especially problematic in tomato plants (*Lycopersicon esculentum* Mill.) cultivated in Japan^4^. Various strategies have been developed to control bacterial wilt, such as grafting^5^, biofumigation^6^ and growing resistant crop varieties^7^, but success has been limited due to the high survival capacity of the bacterium in complex environments^8^ and the wide variety of suitable hosts^9^. To control this disease, growers often graft seedlings on resistant rootstocks^10^. However, the resistance of the rootstocks is unstable^2^, and the scion grafted on the rootstock of a highly resistant cultivar can be latently infected with the pathogen^11^. The disease has recently been found to occur even on grafted plants. Therefore, effective methods for suppressing bacterial wilt are needed.

Various non-pesticide chemicals can be applied in the field to control bacterial wilt because they are less harmful to the environment; however, economic considerations often influence the selection of the chemicals for application. Expensive chemicals and repeated applications are only feasible for valuable crops that may incur substantial economic losses in the absence of treatments. Since the crop yield and quality are not affected when the disease severity is low or the pathogens are absent, a diagnosis based on an economic threshold is essential for determining whether chemical treatments are needed.

Recently, we developed an Fe-polyphenol catalyst using coffee grounds as a raw material, and in a previous study, we demonstrated that this catalyst can be used as an iron fertilizer in agriculture^12,13^ and in the Fenton process to disinfect pathogens such as *E*. *coli*^14^ or to remove methylene blue from water systems^15^. In those works, we proposed that the generation of hydroxyl radicals was responsible for the desired effects. The present study aims to test the application of the Fe-polyphenol catalyst with hydrogen peroxide (H_2_O_2_) to soil as a new method for controlling the soil-borne disease caused by *Ralstonia solanacearum* and to test the involvement of hydroxyl radicals in this process.

## Results

### Soil-borne disease assessment

**Figure 1:**
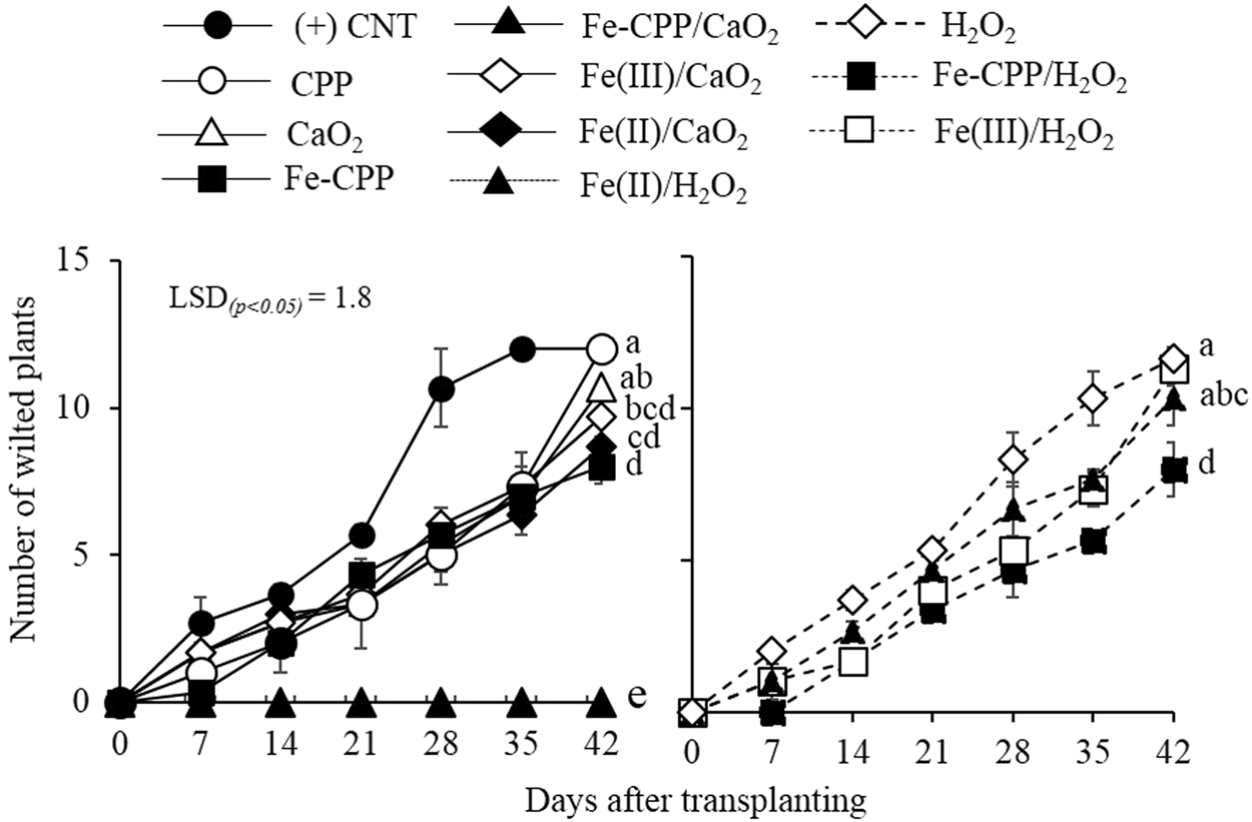
Effect of soil treatments on the incidence of wilt disease caused by *Ralstonia solanacearum* in tomato plants (cv. Momotaro). (+) CNT = positive control, which did not receive any material; CaO_2_ = calcium peroxide (16% W/W); H_2_O_2_ = hydrogen peroxide (35% W/W); Fe-CPP = Fe-polyphenol catalyst developed using coffee grounds; Fe(III) = anhydrous iron (III) chloride; Fe(II) = iron (II) sulfate heptahydrate. All soils were artificially inoculated with *Ralstonia solanacearum* (5.0 log CFU g^−1^ dry soil). Experimental conditions: 4.42 mmol H_2_O_2_ kg^−1^ dry soil as powdered CaO_2_ (16% W/W) or liquid H_2_O_2_ (35% W/W), 1.5 mmol Fe kg^−1^ dry soil as Fe-CPP, Fe(III) or Fe(II) catalysts. Mean values at 42 days after transplantation followed by different letters are significantly different at a *p* < *0*.*05* probability level according to a least significant difference (LSD) test. Bars = standard errors.

**Figure 2:**
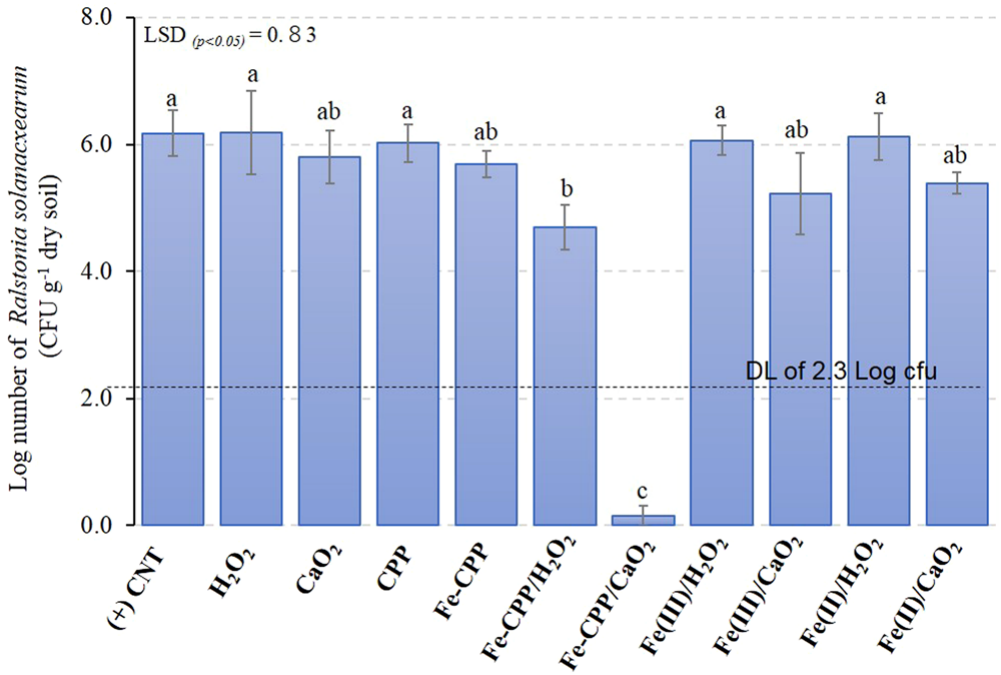
Effect of the treatments on the population of *Ralstonia solanacearum* in the soil after the growth period. CaO_2_ = calcium peroxide (16% CaO_2_) (2 g kg^−1^ dry soil); CPP = coffee polyphenols applied in the form of coffee grounds (2 g kg^−1^ dry soil); Fe-CPP = Fe-polyphenol catalyst developed using coffee grounds (2 g kg^−1^ dry soil); Fe(II) = iron (II) sulfate heptahydrate (0.18 g kg^−1^ dry soil); Fe(III) = anhydrous iron (III) chloride (0.36 g kg^−1^ dry soil). Experimental conditions: 4.42 mmol H_2_O_2_ kg^−1^ dry soil as powdered CaO_2_ (16% W/W) or liquid H_2_O_2_ (35% W/W); 1.5 mmol Fe kg^−1^ dry soil as Fe-CPP, Fe(III) and Fe(II) catalysts. DL = detection limit of the applied selective growth medium. Mean values followed by different letters are significantly different at a *p* < *0*.*05* probability level according to a least significant difference (LSD) test. Bars indicate the standard errors.

The incidence of wilting in the tomato plants during the experimental period differed depending on the material applied. As shown in Fig. 1, the application of Fe-CPP, Fe(III) or Fe(II) with liquid H_2_O_2_ did not completely prevent wilt disease. The disease incidence was markedly higher in the (+) CNT (control) treatment, which was inoculated with the bacteria and did not receive any treatment material. On the other hand, a significant (*p* < *0*.*05*) suppression of the incidence of wilt disease was observed for the Fe-CPP and Fe-CPP/H_2_O_2_ treatments. In addition, complete prevention was observed in the Fe-CPP/CaO_2_ treatment. No significant (*p* < *0*.*05*) decreases in the incidence of the disease were found between the H_2_O_2_, (+) CNT, Fe(II)/H_2_O_2_, Fe(III)/H_2_O_2_, CPP and CaO_2_ treatments. The Fe-CPP/CaO_2_ treatment significantly reduced (*p* < *0*.*05*) the *R*. *solanacearum* population to values below the detection limit of 2 × 10^−2^ CFU g^−1^ dry soil for the used selective medium^16^. No colonies of *R*. *solanacearum* were detected in the autoclaved soil from the (−) CNT treatment. Supplementary Fig. S1 shows a comparison between the two H_2_O_2_ sources applied in conjunction with the Fe-CPP catalyst. The Fe-CPP/H_2_O_2_ treatment resulted in more plants with visible symptoms of wilt disease than the Fe-CPP/CaO_2_ treatment, in which no wilted plants were observed. The *R*. *solanacearum* population in the Fe-CPP/H_2_O_2_-treated soil was 24% lower than that of the (+) CNT-treated soil, while that of the Fe-CPP/CaO_2_-treated soil was 97% lower (Fig. 2). No significant differences were observed between the populations following all other treatments.

### Reactive oxygen species (ROS) assay

**Figure 3:**
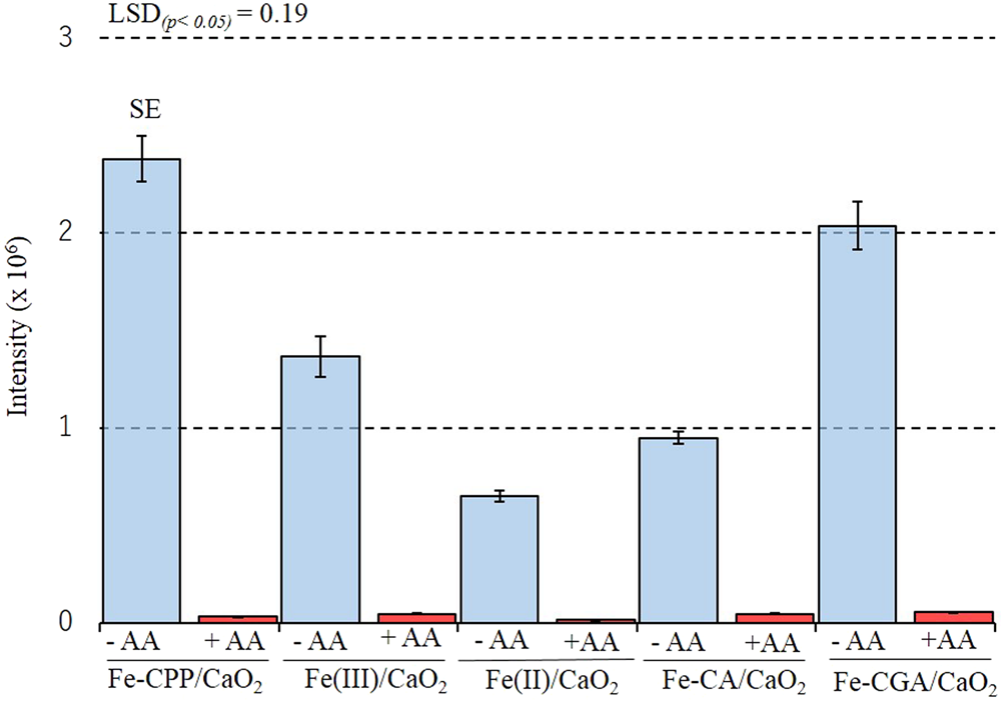
Total reactive oxygen species generated and the effect of the radical scavenger L-ascorbate on the total chemiluminescence intensity of luminol in the CaO_2_ systems. AA = L-ascorbate; CaO_2_ = calcium peroxide (16% CaO_2_); Fe-CPP = Fe-polyphenol catalyst developed using coffee grounds; Fe(II) = iron (II) sulfate heptahydrate; Fe(III) = anhydrous iron (III) chloride; Fe-CA = Fe-caffeic acid complex; Fe-CGA = Fe-chlorogenic acid complex. Conditions: 50 µL of luminol solution (0.13 mol L^−1^ NaOH, 5.4 mmol L^−1^ CaO_2_ and 2.8 mmol L^−1^ Luminol), 50 µL of catalyst solution (1.5 mmol L^−1^ Fe as Fe(II), Fe(III), Fe-CPP, Fe-CA or Fe-CGA), and 50 µL of 10 mmol L^−1^ L-ascorbate (+AA) if necessary. SE = standard error.

Figure 3 shows the total intensity of luminol during 120 s of reaction in the Fe-CPP/CaO_2_, Fe(II)/CaO_2_, Fe(III)/CaO_2,_ Fe-CA/CaO_2_ and Fe-CGA/CaO_2_ systems (where CA is caffeic acid and CGA is chlorogenic acid) and the effect of L-ascorbate on the scavenging of the generated radicals. The total intensity of luminol followed the sequence Fe-CPP/CaO_2_ > Fe-CGA/CaO_2_ > Fe(III)/CaO_2_ > Fe-CA/CaO_2_ > Fe(II)/CaO_2_. For all systems, the addition of L-ascorbate dramatically reduced the total intensity of luminol.

### Hydroxyl radical assay

**Figure 4:**
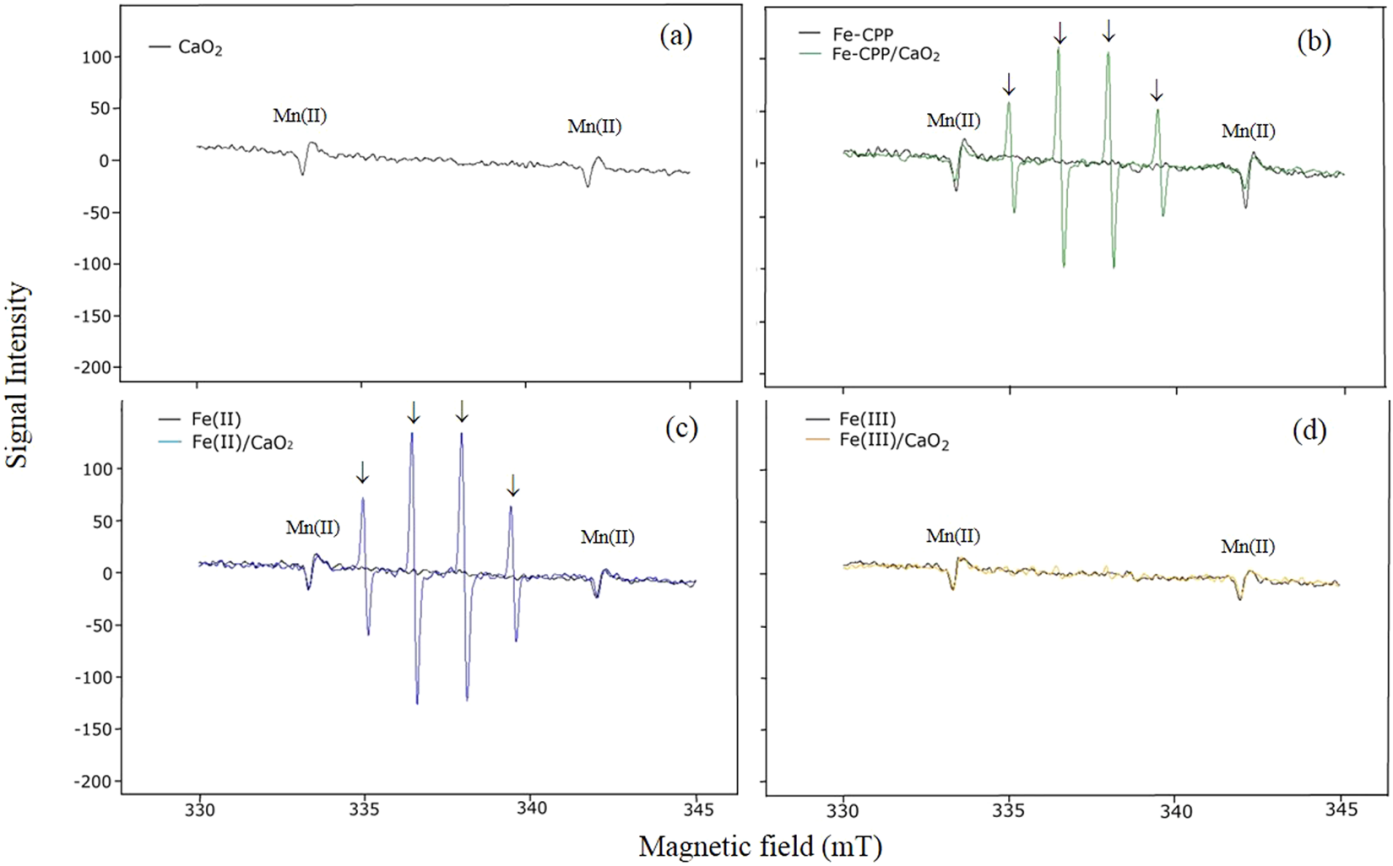
Electron paramagnetic resonance (EPR) spectra of the DMPO-OH radical generated after 30 s of reaction in the CaO_2_ systems. CaO_2_ = calcium peroxide (16%); Fe-CPP = Fe-polyphenol catalyst developed using coffee grounds; Fe(II) = iron (II) sulfate heptahydrate; Fe(III) = anhydrous iron (III) chloride. Reaction conditions: 400 µL of 100 mmol L^−1^ phosphate buffer (pH 7.4); 200 µL of 220 mmol L^−1^ DMPO; 100 µL of 4.42 mmol L^−1^ H_2_O_2_ as CaO_2_ (16% W/W); 100 µL of 1.5 mmol L^−1^ of Fe as Fe-CPP, Fe(III) and Fe(II). The reactions were carried out at room temperature. The peaks associated with the presence of the DMPO-OH radical are indicated with ↓. DMPO = 5,5-dimethyl−1-pyrroline-N-oxide.

**Figure 5:**
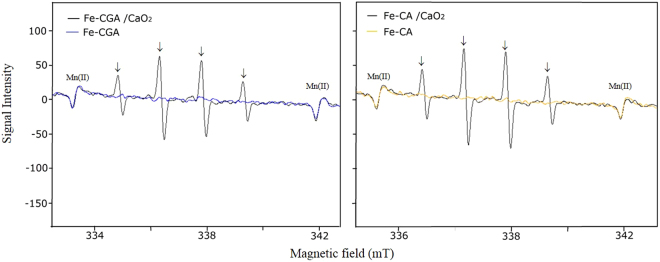
Electron paramagnetic resonance (EPR) spectra of the DMPO-OH radical after 30 s of reaction in the CaO_2_ model systems. CaO_2_ = calcium peroxide; Fe-CA = Fe-caffeic acid complex; Fe-CGA = Fe-chlorogenic acid complex. Reaction conditions: 400 µL of 100 mmol L^−1^ phosphate buffer (pH 7.4); 200 µL of 220 mmol L^−1^ DMPO; 100 µL of 4.42 mmol L^−1^ of H_2_O_2_ as CaO_2_ (16% W/W); 100 µL of 1.5 mmol L^−1^ of Fe as Fe-CPP, Fe(III) and Fe(II). The reactions were carried out at room temperature. The peaks associated with the presence of the DMPO-OH radical are indicated with ↓.

**Figure 6:**
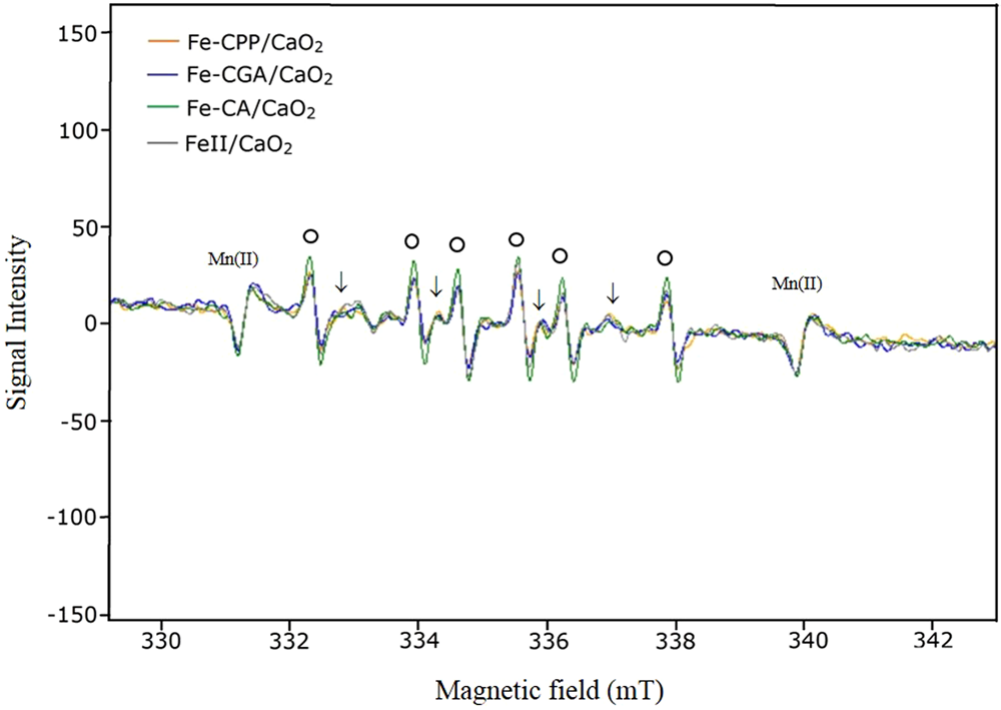
Electron paramagnetic resonance (EPR) spectra of DMPO-CH_3_ after 30 s of reaction in the CaO_2_ systems. CaO_2_ = calcium peroxide (16%); Fe-CPP = Fe-polyphenol catalyst developed using coffee grounds; Fe(II) = iron (II) sulfate heptahydrate; Fe-CA = Fe-caffeic acid complex; Fe-CGA = Fe-chlorogenic acid complex. Reaction conditions: 400 µL of 100 mmol L^−1^ phosphate buffer (pH 7.4); 200 µL of 220 mmol L^−1^ DMPO; 100 µL of 4.42 mmol L^−1^ of H_2_O_2_ as CaO_2_ (16% W/W); 100 µL of 1.5 mmol L^−1^ of Fe as Fe-CPP, Fe(III) and Fe(II); 100 µL of 14.0 mol L^−1^ DMSO solution. Reactions were carried out at room temperature. The peaks associated with the presence of the DMPO-OH radical are indicated with ↓, and those associated with DMPO-CH_3_ are indicated with ○.

**Figure 7:**
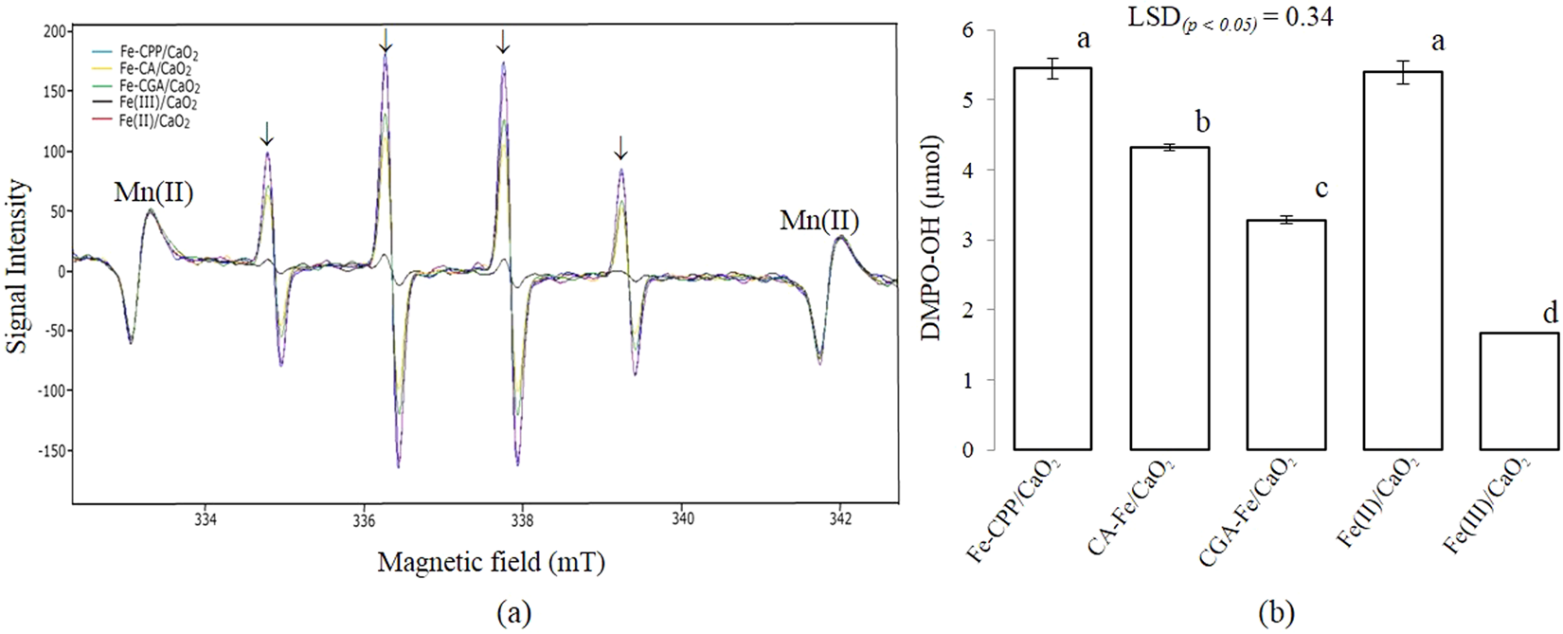
Generation of Hydroxyl radicals by different catalysts in the powdered CaO_2_ systems. (**a**) Electron paramagnetic resonance (EPR) spectra and (**b**) yield of DMPO-OH generated after 30 s of reaction in the powdered CaO_2_ systems. Fe-CPP = Fe-polyphenol complex developed using coffee grounds, Fe(II) = iron (II) sulfate heptahydrate; Fe(III) = iron (III) chloride anhydrous, Fe-CA = Fe-caffeic acid complex, Fe-CGA = Fe-chlorogenic acid complex. Reaction conditions: 400 µL of 100 mmol L^−1^ phosphate buffer (pH 7.4), 200 µL of 220 mmol L^−1^ DMPO, 100 µL of 4.42 mmol L^−1^ H_2_O_2_ as powdered CaO_2_ (16% W/W) and 100 µL of 1.5 mmol L^−1^ Fe as Fe-CPP, Fe(III), Fe(II), F-CA or Fe-CGA. 4-Hydroxy-2,2,6,6-tetramethylpiperidine-N-oxyl (TEMPOL) was used as the standard to calculate the concentrations of DMPO-OH. Reactions were carried out at room temperature. Peaks associated with the presence of DMPO-OH radical are indicated with ↓. Mean values followed by different letters are significantly different at a *p* < *0*.*05* probability level according to a least significant difference (LSD) test. Bars indicate the standard errors.

The results of the electron paramagnetic resonance (EPR) experiments are shown in Figs 4–7. The presence of the 5,5-dimethyl-1-pyrroline-N-oxide (DMPO)-OH radical was confirmed by the observed hyperfine coupling constants (hfcc) of aN = aH = 1.49 mT^17^. Figure 4 shows the spectra of the DMPO-OH radical after 30 s of reaction in the following systems: (a) CaO_2_, (b) Fe-CPP/CaO_2_ and Fe-CPP, (c), Fe(II)/CaO_2_ and Fe(II), and (d) Fe(III)/CaO_2_ and Fe(III). Systems that not received liquid or powdered CaO_2_ as H_2_O_2_ no signals of DMPO-OH radical were detected. On the other hand, CaO_2_ and Fe(III)/CaO_2_ systems showed DMPO-OH radical signals among the treatments that received liquid or powdered CaO_2_ as H_2_O_2_ source. The signals characteristics of the DMPO-OH radical were also detected in the Fe-CA/CaO_2_ and Fe-CGA/CaO_2_ model systems (Fig. 5). When dimethyl sulfoxide (DMSO) was added to the reaction systems, DMPO-CH_3_ (the spin adduct of methyl radical, hfcc: aN = 1.64 mT, aH = 2.35 mT)^18^ was observed, and the intensity of the signals for the DMPO-OH radical decreased (Fig. 6). Figure 7 shows the EPR spectra and the yield of the DMPO-OH radical generated after 30 s of reaction in the powdered CaO_2_ systems. Quantitative analysis revealed that the yields of the DMPO-OH radical generated by CPP-Fe/CaO_2_ were 1.3-, 1.7- and 3.3-fold higher than those generated by the Fe-CA/CaO_2_, Fe-CGA/CaO_2_ and Fe(III)/CaO_2_ systems, respectively. However, no differences (*p* < *0*.*05*) were found between the amounts of the DMPO-OH radical generated after 30 s of reaction time in the Fe-CPP/CaO_2_ and the Fe(II)/CaO_2_ systems. The amount of hydroxyl radical generated after 30 s of reaction time followed the order Fe-CPP = Fe(II) > Fe-CA > Fe-CGA >> Fe(III).

## Discussion

In previous experiments, the results of an XPS survey revealed that both ferric iron (Fe^3+^) and ferrous iron (Fe^2+^) were present in the Fe-polyphenol catalyst but no zerovalent iron (nZVI) was present. Iron was present in the forms of Fe_2_O_3_/FeCl_2_ and FeCl_3_^14^. On the other hand, more than 98% of the iron released from the Fe-polyphenol catalyst was in the Fe^2+^ form as detected by the phenanthroline method^15^. The results of *in vitro* experiments showed that the Fe-polyphenol catalyst can be used to supply iron to leaf vegetables^12^ and rice^13^, and *in vitro* experiments under laboratory conditions showed that when applied in conjunction with liquid H_2_O_2_, this catalyst could disinfect pathogens such as *Escherichia coli*^14^ and *Ralstonia solanacearum* (see Supplementary Figs S2, S3, and S4) or remove methylene blue from water systems^15^. We proposed a mechanism involving the generation of hydroxyl radicals by the reaction between the iron catalyst and H_2_O_2_. In the present study, the same Fe-polyphenol catalyst was prepared and applied with two H_2_O_2_ sources with different H_2_O_2_ release rates to suppress the bacterial wilt disease caused by *R*. *solanacearum*, which is one the most difficult soil-borne disease to control because the bacteria can survive in various environments^19,20^.

A chemiluminescence method based on luminol was used to verify the presence of ROS^21^ by adding L-ascorbate, which can scavenge ROS, decreasing the chemiluminescence intensity. A high chemiluminescence intensity was found for all treatments, and the addition of L-ascorbate dramatically reduced the emission intensity of luminol, indicating the presence of radical species in all systems. The high luminol intensity observed in the Fe-CGA/CaO_2_ and Fe-CA/CaO_2_ model systems suggests that chlorogenic acid and caffeic acid may be associated with the generation of ROS in the Fe-CPP/CaO_2_ system since these acids are the predominant polyphenols found in coffee grounds^22–25^. Luminol is a good indicator of the presence of ROS but cannot identify specific radicals because it emits chemiluminescence with all kinds of radicals, such as ·OH, ·O_2_^−^ and ^1^O_2_. Hydroxyl radicals are the most reactive and least selective ROS^26^, and they could play a role in the results of this experiment. To test this hypothesis, a series of EPR experiments using DMPO as a spin trap were carried out. The results are shown in Figs 4–7. No signals for DMPO-OH radicals were detected in the systems without an added H_2_O_2_ source (Figs 4 and 5). In addition, as shown in Fig. 6, when DMSO was added to the reaction systems in which the DMPO-OH radical was detected, a signal for the DMPO-CH_3_ radical was observed, and the intensity of the DMPO-OH radical signal decreased.

DMPO-CH_3_ is produced through the oxidation of DMSO by hydroxyl radicals, indicating that the DMPO-OH radical signal detected by EPR analysis represents the generation of hydroxyl radicals^27,28^ rather than the nucleophilic addition of water^29^. Thus, coffee grounds might contain polyphenols that can contribute to the generation of hydroxyl radicals when bound to iron as a catalyst in the Fenton process. In addition, the hydroxyl radicals generated by the modified Fenton system using the Fe-CPP catalyst might contribute to the lethal oxidative damage to the bacterial cells^30^ occurring in the studied soil. These results show that hydroxyl radicals were the major ROS in the Fe-CPP/CaO_2_ and Fe(II)/CaO_2_ systems and agree with those showing that hydroxyl radicals are the major ROS in Fe(II)/CaO_2_ systems^31^.

The present study demonstrated that the generation of hydroxyl radicals by the reaction of CaO_2_ with an Fe-polyphenol catalyst developed using coffee grounds was associated with the observed bactericidal effects. Hydroxyl radicals have the highest oxidation potential (2.76 V) among ROS and are generated in the reaction between iron (II) as a catalyst and H_2_O_2_ as an oxidant^32^. The disease incidence was drastically reduced by the Fe-CPP/CaO_2_ treatment compared to the Fe-CPP/H_2_O_2_ treatment. This effect remained until the fruiting stage (see Supplementary Fig. 4S). These results agree with recent studies suggesting that CaO_2_ is a more effective source of H_2_O_2_ than liquid H_2_O_2_ for *in situ* chemical oxidation^33–35^. The chemical oxidation capacity of CaO_2_ is dependent on the generation of H_2_O_2_ (equation (1)) and the subsequent production of hydroxyl radicals from the released H_2_O_2_ (equation (2))^36,37^.

1 $${{\rm{CaO}}}_{2}+2{{\rm{H}}}_{2}{\rm{O}}\to {{\rm{H}}}_{2}{{\rm{O}}}_{2}+{\rm{Ca}}{({\rm{OH}})}_{2}$$

2 $${{\rm{H}}}_{2}{{\rm{O}}}_{2}+{{\rm{Fe}}}^{2+}\to \cdot \,{\rm{OH}}+{{\rm{OH}}}^{-}+{{\rm{Fe}}}^{3+}$$

The advantage of this reaction is that the concentration of released H_2_O_2_ is autoregulated by the rate of CaO_2_ dissolution, which reduces the disproportionation of H_2_O_2_ in the media since not all the H_2_O_2_ is available at once, as is the case with liquid H_2_O_2_^38^. In our experiments, the lower efficacy of liquid H_2_O_2_ compared with that of powdered CaO_2_ as a source of H_2_O_2_ was obvious and could be explained through the rapid decomposition of liquid H_2_O_2_ that occurs in soils. These factors limit the applicability of the modified Fenton process for *in situ* chemical oxidations^35^. The most important limitation of the conventional Fenton reagent is the instability of the large amount of hydroxyl radicals instantaneously produced from liquid H_2_O_2_^34,35^. The excess H_2_O_2_ could act as a scavenger and compete for hydroxyl radicals^39,40^, inhibiting the oxidation of bacterial cells. In this study, the release of H_2_O_2_ was autoregulated by the rate of CaO_2_ dissolution, which prevented all the H_2_O_2_ from being available at once, as it is when liquid H_2_O_2_ is used as the reagent^34^. As a result, the bactericidal effect of the H_2_O_2_ reaction with Fe-polyphenol increased when CaO_2_ was used. On the other hand, the amount of hydroxyl radicals produced by the Fe-polyphenol-activated CaO_2_ was estimated to be much higher than that generated by the Fe(II) or Fe(III) catalysts, which was verified by EPR spectroscopy (Fig. 7).

In our experiment, the failure of the Fe(II) and Fe(III) catalysts to reduce the incidence of wilt disease when applied with either source of H_2_O_2_ was studied (Fig. 1). These results can be explained by the lower total radical concentration produced by the Fe(III)/CaO_2_ and Fe(II)/CaO_2_ systems than that produced by the Fe-CPP/CaO_2_ treatment. The weak effect of the Fe(III)/CaO_2_ treatment on wilt disease could be attributed to the low reactivity of Fe(III) with H_2_O_2_, which results in a lower content of OH radicals produced. Compared to other catalysts, the Fe(III) catalyst produced a lower yield of hydroxyl radicals when reacted with the same amounts of H_2_O_2_ and powdered CaO_2_ (Fig. 7). The Fe(III)-activated CaO_2_ exhibited several limitations, such as precipitation of the iron as ferric hydroxide (Fe(OH)_3_), which does not readily redissolve and inhibits the oxidation process^41^. The addition of chelating agents such as citric acid, tartaric acid, oxalic acid, and glutamic acid has been proposed as a way to overcome these drawbacks^41,42^. We believe that the caffeic acid and chlorogenic acid present in coffee grounds probably contributed to the Fenton process by reducing Fe^3+^ to Fe^2+^ and/or served as electron donors binding Fe^2+^ to maintain the activity of Fe in the reduced state in the Fenton cycle.

A single application of H_2_O_2_ to the soil did not reduce the disease incidence. Usually, a solution containing 588 to 3529.4 mmol L^−1^ H_2_O_2_ is used in the *in situ* chemical oxidation process^43^, but the half-life of H_2_O_2_ at these concentrations is only minutes to hours. These degradation rates are much higher than that of the 1.5 mmol L^−1^ H_2_O_2_ solution used in this experiment.

For the *in situ* chemical oxidation process, iron can be added as Fe^2+^ or Fe^3+^ salts^44^ or as native iron-containing minerals such as goethite and ferrihydrite^45,46^. The low solubility of Fe^3+^ at neutral pH necessitates the use of chelators to increase the Fe^3+^ concentration in the aqueous phase^47,48^. Citric acid, oxalic acid, ethylenediaminetetraacetic acid, 1,4-benzenedicarboxylic acid, N,N-dimethyl formamide and tartaric acid have been successfully applied as Fe^3+^ chelating agents for the Fenton process^36,49^. If insufficient Fe^2+^ is added or if only Fe^3+^ is present initially, Fe^2+^ is regenerated through various reactions^50^.

Our results are consistent with those of other studies^27,51^. The detected EPR signals together with the results of the scavenging tests with L-ascorbate indicated that hydroxyl radicals were the major ROS in the Fe-CPP/CaO_2_, Fe-CA/CaO_2_, Fe-CGA/CaO_2_ and Fe(II)/CaO_2_ systems but not in the Fe(III)/CaO_2_ system, as no DMPO-OH radical signal was detected in this system. The peaks of O·_2_^−^ were not confirmed in the EPR analyses of all the treatments, indicating that low concentrations of O·_2_^−^ were generated in the systems studied.

**Figure 8:**
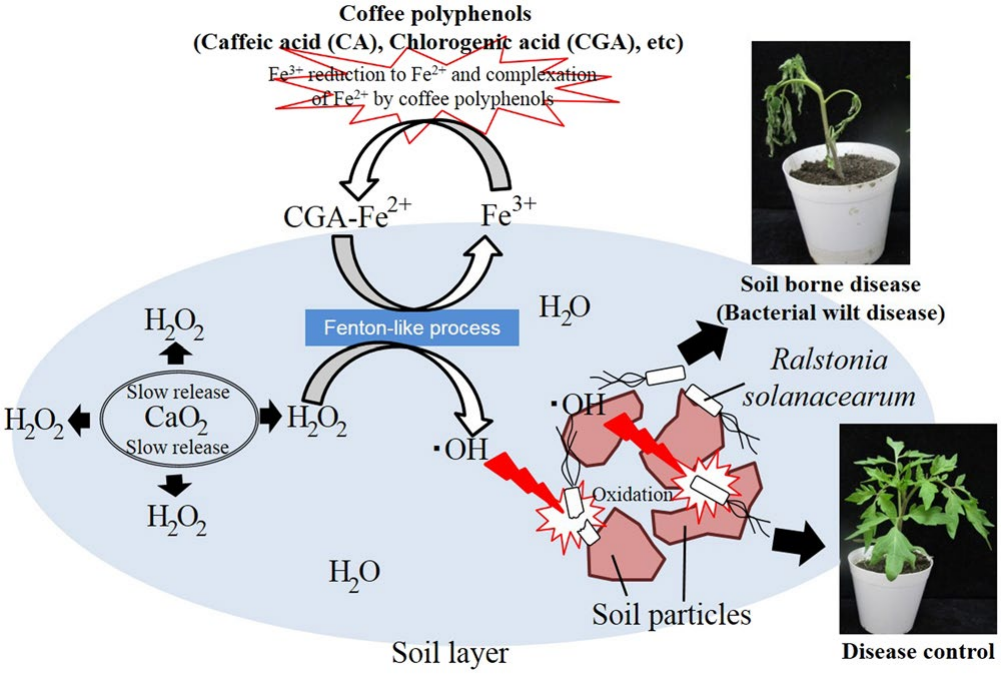
Proposed mechanism of hydroxyl radical (⋅OH) formation for the treatment of soil-borne disease by the Fe-CPP activation of powdered calcium peroxide (CaO_2_).

Figure 8 shows the proposed mechanism for the treatment of soil-borne disease by the CAF-Fe activation of powdered CaO_2_. First, Fe^3+^ is reduced to Fe^2+^, and then the Fe^2+^ forms a complex with the coffee polyphenols. The Fe^2+^-polyphenol species react with the H_2_O_2_ from the calcium peroxide to generate ∙OH radicals. Finally, the ∙OH radicals oxidize the bacterial cells in the soil. We proposed that the coffee polyphenols such as chlorogenic acid and caffeic acid used in our study reduced and chelated the iron, creating conditions that favour the oxidation of bacterial cells in the soil environment by the Fenton process. Generally, hydroxyl radicals are generated from electron transfer between the complex of H_2_O_2_ and iron sites. The electron-rich organic ligands could donate electrons to the Fe ions^51^. Coffee polyphenols probably contributed to the Fenton process by reducing Fe^3+^ to Fe^2+^ and/or served as electron donors to maintain the activity of Fe in its reduced state in the Fenton cycle. Reduction of Fe^3+^ generates Fe^2+^, which can participate in the Fenton reaction and generate ROS^52,53^.

Regardless of the investigated Fe-polyphenols and CaO_2_ as an advancement in soil-borne disease control, further investigations are required to evaluate the injection mode of these particles in soils. The developed method could reduce the dependence on high-risk chemicals for disease management, and this method is ecologically sound and environmentally friendly. Evaluating the effectiveness of CPP-Fe/CaO_2_ for controlling soil-borne disease on a large scale is difficult because few controlled studies on the rate of dissolution of CaO_2_ and the yield of H_2_O_2_ in different types of soil and on the stability of the CPP-Fe material in soil have been reported. The efficiency of the treatment will significantly depend on the contact between the bacteria and the catalyst with the CaO_2_ particles. Therefore, particles with a high mobility must easily reach the contaminated target soil layers. Other factors such as soil pH, natural scavengers, soil texture, and water content could alter the effectiveness of Fe-polyphenol-activated CaO_2_ for controlling soil-borne disease in field conditions. The release rate of H_2_O_2_ from CaO_2_ is autoregulated by the rate of CaO_2_ dissolution, which can be controlled by adjusting the pH^54^. Carbonate and bicarbonate buffer species act as radical scavengers in the Fenton process^55^. Thus, the soil pH could certainly alter the effectiveness of the CPP-Fe/CaO_2_ treatment. The carboxylate or phenolic functional groups in natural organic substances could act as a ligand for Fe(II), scavenge hydroxyl radicals, or reduce ferric oxides altering the effectiveness of Fenton or Fenton-like reactions^56^. Humic acid can act as a free-radical scavenger, as a radical chain promoter, and as a catalytic site inhibitor^56,57^. Fenton oxidation and ·OH production were enhanced in the presence of peat by one or more peat-dependent mechanisms^58^. The Fe concentration and availability in the peat, the reduction of Fe^3+^ to Fe^2+^ by the organic matter, and the reduction of organic-complexed Fe^3+^ to Fe^2+^ were probable causes of this enhancement. In addition, microbial activity may also be responsible for hydrogen peroxide decomposition^59^.

The presence of inorganic components in the soil could affect the generation of ·OH. Ammonium sulfate and monobasic sodium phosphate have been used to stabilize hydrogen peroxide^60^. Of the four inorganic stabilizers (i.e., monobasic potassium phosphate, dibasic potassium phosphate, sodium tripolyphosphate, and silicic acid) for hydrogen peroxide, monobasic phosphate was found to propagate hydrogen peroxide over the longest distance in soil columns^61^; however, monobasic phosphate was depleted by adsorption and may also function as a radical scavenger^60^. Those stabilizers could increase the effectiveness of the CPP-Fe/CaO_2_ treatment.

The mobility of the Fe-CPP and CaO_2_ particles in soils (i.e., saturated and unsaturated zones) should be investigated prior to *in situ* applications. The effect of Fe-CPP/CaO_2_ treatment on soil quality and native microbiota should be investigated. Prior to field or *in situ* applications, feasibility studies are necessary to determine the extent and rate of bacterial oxidation on a batch scale.

## Conclusion

From the results obtained in this work, we conclude that the polyphenols in coffee, such as caffeic acid and chlorogenic acid, play an important role in the generation of hydroxyl radicals in the Fe-polyphenol catalyst developed using coffee grounds. The developed catalyst is low-cost, has a low toxicity and could be used as an environmentally friendly method for suppressing the incidence of soil-borne diseases. However, the feasibility of this method on the field scale needs to be verified.

## Material and Methods

### Chemicals

2,3,5-Triphenyl tetrazolium chloride and 5,5-dimethyl-1-pyrroline-N-oxide (DMPO) were purchased from Tokyo Chemical Industry Co., Japan. Anhydrous iron (III) chloride was obtained from Kanto Chemical, Japan. H_2_O_2_ (35% W/W), agar (powder), chloramphenicol, crystal violet, cycloheximide, polymyxin B sulfate, calcium peroxide (CaO_2_), caffeic acid (CA, 3,4-dihydroxycinnamic acid), chlorogenic acid (CGA), iron(II) sulphate and phosphate buffer (pH 7.4) were purchased from Wako Pure Chemical Industries, Japan. Casamino acids, peptone and dextrose were purchased from Becton Dickinson and Co., Sparks, United States. Other chemicals were of reagent grade and were used as received without further purification. Coffee grounds were collected from a coffee beverage company (AGF Co., Suzuka, Japan).

### Synthesis of iron catalysts

Eighty-eight grams of coffee grounds was mixed with 12 g of anhydrous iron (III) chloride (Fe(III)) and 300 mL of water. The mixture was heated to 98 °C for 24 hours and then dried at 82 °C for 48 hours. The coffee grounds-iron mixture was subsequently ground before the experiments^15^.

CA and CGA, which are the main polyphenols in coffee^22–25^, were reacted with iron and used as Fe-polyphenol models to clarify the role of these Fe-polyphenol complexes in the activation of CaO_2_ and the generation of hydroxyl radicals. The Fe-CGA and Fe-CA complexes were prepared with deionized water. A total of 252.2 mg of CA and 496.0 mg of CGA were individually mixed with 227.1 mg L^-1^ of anhydrous iron (III) chloride (Fe(III)).

Iron (II) sulfate heptahydrate (Fe(II)) and Fe(III) chloride (Fe(III)) catalysts were used as pure salts.

### Soil-borne disease assessment

Tomato cv. Momotaro was used as the test specie. For the inoculum, *Ralstonia solanacearum* MAFF301487^4^ (see Supplementary Fig. S5) was cultured in 1 L of casamino acid-peptone-glucose medium (CPG medium) (0.1% casamino acid, 1% peptone, and 0.5% glucose, pH 7.0) in a sealed 500 mL Erlenmeyer flask at 32 $^\circ \,$C for 3 days in the dark with continuous shaking. All treatments, except for the negative control treatment ((−) CNT), were inoculated with this bacterial solution.

Two hundred and fifty grams of previously sterilized gardening soil (NIPPI, Nihon Hiryo Co., Tokyo, Japan) was placed in a polyethylene plant pot (9.2 cm × 8.2 cm, Asahikasei, Tokyo, Japan) and inoculated with the bacterial solution to a final *R*. *solanacearum* population of 5.0 log CFU g^−1^ dry soil. Then, the following treatments were applied: no inoculation of an *R*. *solanacearum* treatment: 1. negative control: no application of any material ((−) CNT); inoculation of *R*. *solanacearum* treatments: 2. positive control: no application of any material ((+) CNT); 3. 300 mL of 1.5 mmol L^−1^ liquid H_2_O_2_ (H_2_O_2_); 4. powdered CaO_2_ (16% W/W); 5. coffee polyphenols from coffee grounds (CPP); 6. Fe-polyphenol catalyst developed using coffee grounds (Fe-CPP); 7. Fe-CPP and liquid H_2_O_2_ (Fe-CPP/H_2_O_2_); 8. Fe-CPP and powdered CaO_2_ (Fe-CPP/CaO_2_); 9. iron (II) sulfate heptahydrate and liquid H_2_O_2_ (Fe(II)/H_2_O_2_); 10. iron (II) sulfate heptahydrate and CaO_2_ (Fe(II)/CaO_2_); 11. anhydrous iron (III) chloride and liquid H_2_O_2_ (Fe(III)/H_2_O_2_); and 12. anhydrous iron (III) chloride and powdered CaO_2_ (Fe(III)/CaO_2_). Both the liquid H_2_O_2_ (35% W/W) and powdered CaO_2_ (16% W/W) treatments were applied at the same final concentrations (4.42 mmol H_2_O_2_ kg^−1^ dry soil). The catalysts Fe-CPP, iron (II) sulfate heptahydrate (Fe(II)) and iron (III) chloride anhydrous (Fe(III)) were applied at the same final concentrations (1.5 mmol Fe kg^−1^ dry soil) in their respective treatments. Each treatment was repeated three times (twelve pots per replicate) with one plant per pot. The disease incidence was assessed by counting the wilting plants at weekly intervals for 42 days postinoculation. The populations of *R*. *solanacearum* in the soils at the end of the experiment were estimated using a selective medium^16^. Tomato seeds were sown in a tray, and the seedlings were transplanted when they reached 10 cm in height. The soil moisture level does not affect *Ralstonia solanacearum* populations except in instances of severe drought. To minimize the effect of drought on the bacterial populations, water was continuously provided by placing the pots in a tray in which the water level was maintained at 5 mm from the bottom by frequent watering.

### Reactive oxygen species (ROS) assay

A chemiluminescence assay^62^ was carried out to determine the total amount of ROS generated in the reaction of CaO_2_ with the Fe-CPP, Fe(II), Fe(III), Fe-CA and Fe-CGA catalysts. Fifty microlitres of each iron catalyst solution containing 1.5 mmol L^−1^ of Fe was transferred to a tube and placed in a luminometer (AB 2270, ATTO, Tokyo, Japan), and then, 50 μL of a solution containing 0.13 mol L^−1^ of NaOH, 4.42 mmol L^−1^ H_2_O_2_ in the form of CaO_2_ and 2.8 mmol L^−1^ luminol was injected into the system via a pump through the upper injection port. Fifty microlitres of 10 mmol L^−1^ L-ascorbate was added to the reaction to verify the presence of radicals. The intensities of the signals were recorded for 120 s.

The H_2_O_2_ in the samples was analysed by a spectroscopic method^63^ using a UV spectrophotometer (UV-1800, Shimadzu, Tokyo, Japan).

### Hydroxyl radical (·OH) assay

An EPR assay was carried out to identify the presence of hydroxyl radicals in the systems. To follow the hydroxyl radical generation in the modified Fenton reaction using the iron catalysts, a spin trapping method using DMPO was employed. In the spin trapping experiment, 400 µL of phosphate buffer (pH 7.4) was mixed with 200 µL of 220 mmol L^−1^ DMPO, 100 µL of 4.42 mmol L^−1^ H_2_O_2_ in the form of liquid H_2_O_2_ (35% W/W) or CaO_2_ (16% W/W) and 100 µL of 1 mmol L^−1^ Fe in the form of Fe-CPP, Fe(III) and Fe(II). To investigate whether the observed DMPO-OH radical originated from hydroxyl radical generation, an additional assay was performed in which 100 μL of 14 mol L^−1^ DMSO, an authentic hydroxyl radical scavenger, was added to each reaction system. Furthermore, the reactions of Fe-CGA and Fe-CA with CaO_2_ were performed as models. The EPR spectra were recorded 30 s after the addition of the respective iron catalyst using an X-band EPR spectrometer (MS 5000, Magneteck, Berlin, Germany). The measurement conditions for EPR were as follows: magnetic field, 337.5 mT; field modulation frequency, 100 kHz; field modulation width, 0.16 mT; sweep time, 60 s; microwave frequency, 9.463 GHz; and microwave power, 5 mW.

### Statistical analyses

Completely randomized designs were used in all the experiments. Statistical significance (*p* < *0*.*05*) for the wilt disease assay, population of *R*. *solanacearum* in the soil and total ROS generated were each assessed by one-way analysis of variance (ANOVA) followed by Fisher’s least significant difference (LSD) post hoc test for multiple comparisons at a significance level of *p* < *0*.*05*.

### Data availability

All data generated or analysed during this study are included in this published article (and its Supplementary Information files). The data sheets generated and/or analysed in the current study are available from the corresponding author on reasonable request.

## Electronic supplementary material

Supplementary Information
