# Supplementary material for: Generation of hydroxyl radicals by Fe-polyphenol-activated CaO2 as a potential treatment for soil-borne diseases
Source: Sci Rep. 2018 Jun 27;8:9752. doi: 10.1038/s41598-018-28078-6 (PMC6021405; doi:10.1038/s41598-018-28078-6)
Supplement: Supplementary file 1 — Supplementary Information [file 41598_2018_28078_MOESM1_ESM.doc]

**Generation of hydroxyl radicals by Fe-polyphenol-activated CaO_2_ as a potential treatment for soil-borne diseases**

Cláudio Kendi Morikawa

National Agriculture and Food Research Organization, Division of Vegetable Pest Management and Functional Analysis, Institute of Vegetable and Floriculture Science, 514-2392, Ano, Kusawa 360, Mie, Tsu, Japan. (email:ckm@affrc.go.jp)

**
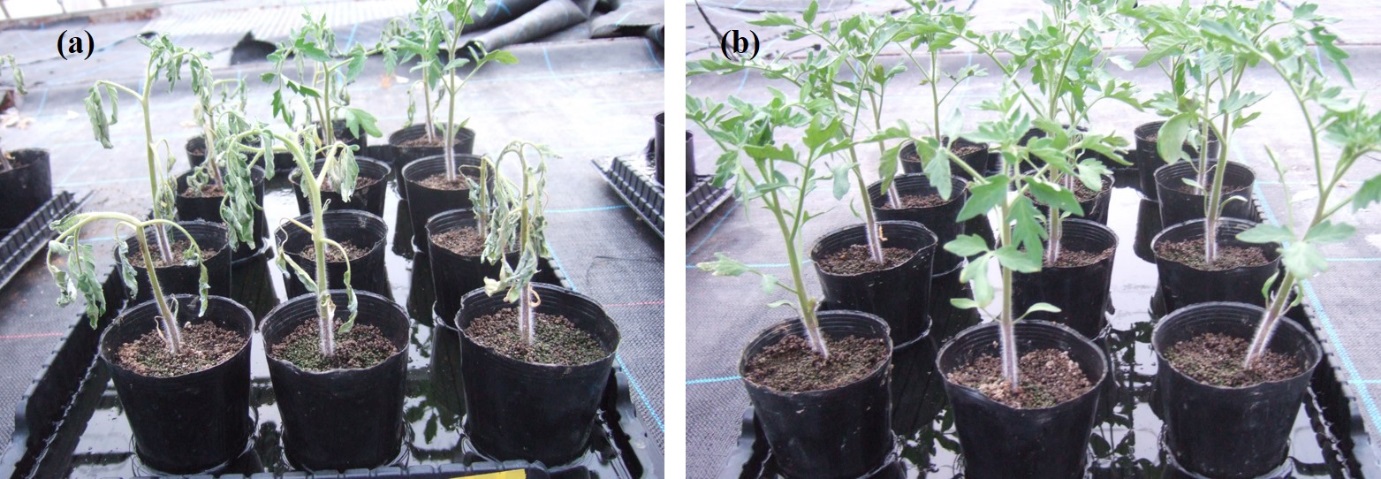
**

**Figure S1. View of the wilt symptoms of the tomato plants (cv. Momotaro).** (a) Fe-CPP/H_2_O_2_ and (b) Fe-CPP/CaO_2_ treatments. Fe-CPP = Fe-polyphenol catalyst developed using coffee grounds; H_2_O_2_ = hydrogen peroxide; CaO_2_ = calcium peroxide.


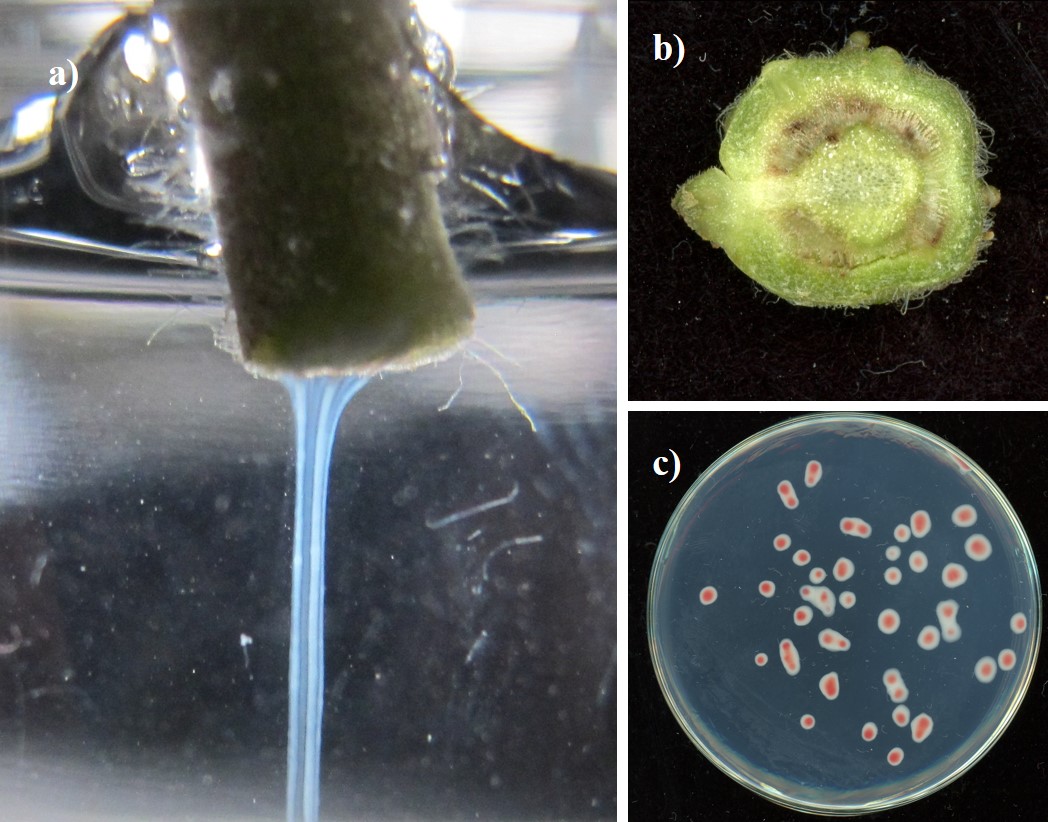


Figure S2. Bacterial wilt disease observed in tomato plants. (a) Bacterial ooze from a cut tomato stem infected with *Ralstonia solanacearum* MAFF301487; (b) brown discoloration of the cross section of the stem of an infected plant; and (c) culture of *R. solanacearum* on a selective medium.


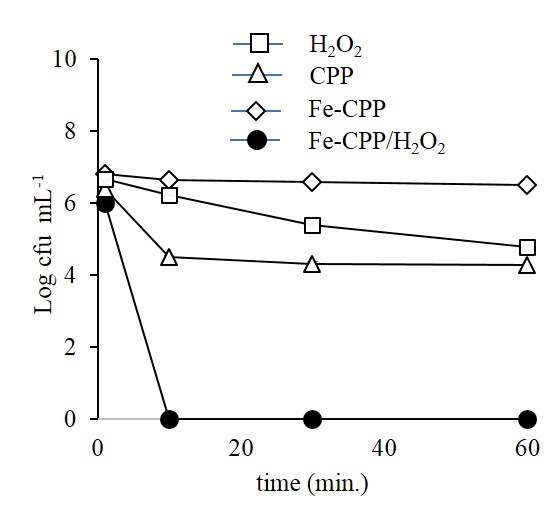


**Figure S3. Effect of Fe-polyphenol and liquid H_2_O_2_ on *R. solanacearum* (*in vitro* experiment).** (a) Single application H_2_O_2_, (b) single application of Fe-CPP and (c) Fe-CPP/H_2_O_2_. CPP = coffee polyphenols applied as coffee grounds (2 g L^-1^ solution); Fe-CPP = Fe-polyphenol catalyst developed using coffee grounds. Experimental conditions: 0.1 mmol L^-1^ Fe as Fe-CPP; 1 mmol L^-1^ H_2_O_2_ as liquid H_2_O_2_; Ralstonia solanacearum (6.0 log CFU L^-1^ solution).


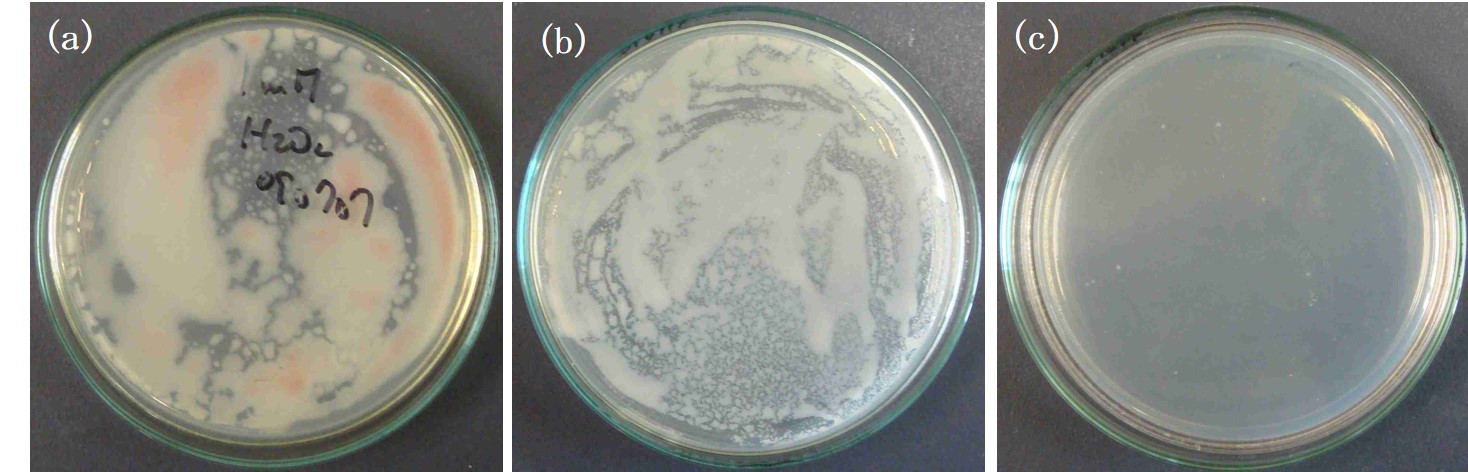


**Figure S4. Culture of *R. solanacearum* on Hara & Ono’s selective agar medium.** (a) Single application of H_2_O_2_, (b) single application of Fe-CPP catalyst and (c) Fe-CPP/H_2_O_2_. Fe-CPP = Fe-polyphenol catalyst developed using coffee grounds. Experimental conditions: 0.1 mmol L^-1^ Fe as Fe-CPP; 1 mmol L^-1^ H_2_O_2_ as liquid H_2_O_2_; *Ralstonia solanacearum* (6.0 log CFU L^−1^ solution).


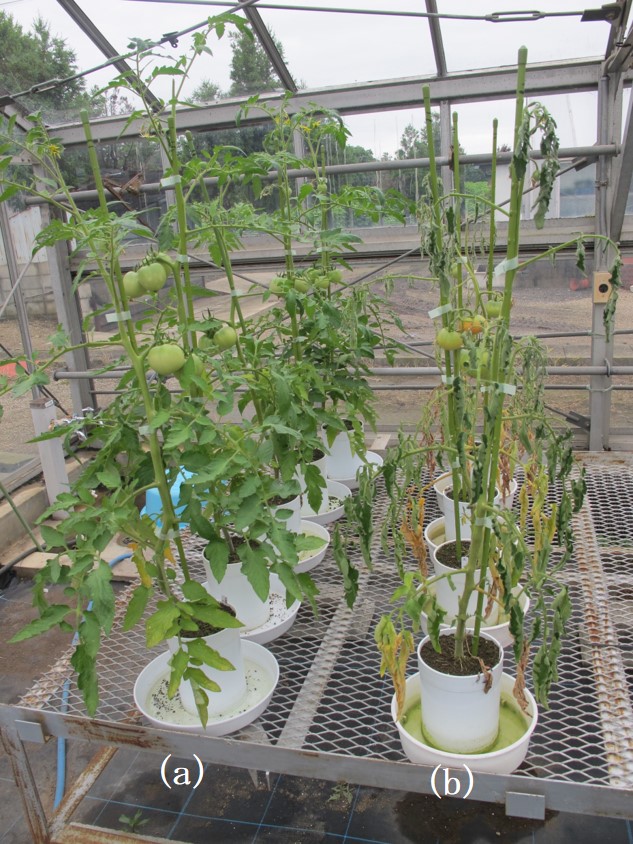


Figure S5. Photograph of plants grown in soil artificially inoculated with *R. solanacearum*. (a) Fe-CPP/CaO_2_ and (b) Fe-CPP/H_2_O_2_. Fe-CPP = Fe-polyphenol catalyst developed using coffee grounds; CaO_2_ = calcium peroxide. Experimental conditions: 0.1 mmol L^-1^ Fe as Fe-CPP; 4.42 mmol L^-1^ H_2_O_2_ as powdered CaO_2_ or liquid H_2_O_2_; *Ralstonia solanacearum* (5.0 log CFU g^−1^ dry soil).
